# Characterization of novel genetic alterations in salivary gland secretory carcinoma

**DOI:** 10.1038/s41379-019-0427-1

**Published:** 2019-12-10

**Authors:** Kiyong Na, Juan C. Hernandez-Prera, Jae-Yol Lim, Ha Young Woo, Sun Och Yoon

**Affiliations:** 1 Department of Pathology, Yonsei University College of Medicine, Yonsei University Health System, Severance Hospital, Seoul, South Korea; 2 Department of Pathology, College of Medicine, Kyung Hee University, Seoul, South Korea; 3 Department of Anatomic Pathology, H. Lee Moffitt Cancer Center and Research Institute, Tampa, FL USA; 4 Department of Otorhinolaryngology, Yonsei University College of Medicine, Yonsei University Health System, Seoul, South Korea

**Keywords:** Cancer genetics, Cancer genomics, Head and neck cancer

## Abstract

Secretory carcinoma is a salivary gland tumor with a characteristic chromosomal translocation that results in an *ETV6-NTRK3* fusion gene. Secretory carcinoma shows relatively frequent rates of lymph-node metastasis and tumor recurrence and has a characteristic histology. Except for the *ETV6* translocation, genomic alterations in secretory carcinoma have not been reported. In the present study, we characterized the novel recurrent genetic mutations of secretory carcinoma. On the basis of histology, immunohistochemistry, and *ETV6* gene break-apart fluorescence in situ hybridization assays, 22 tumors were classified as secretory carcinomas (19 *ETV6* translocation-positive and 3 *ETV6* translocation-negative secretory carcinomas) and their clinicopathologic characteristics were reviewed. Targeted deep sequencing analyses were performed on 20 secretory carcinomas (17 *ETV6* translocation-positive and 3 *ETV6* translocation-negative secretory carcinomas) to investigate their genetic alterations. The A16V (C→T) mutation in *PRSS1*, which encodes a cationic trypsinogen and has a mutation associated with hereditary pancreatitis and pancreatic adenocarcinoma, was observed in 40% (8/20) (7/17 of *ETV6* translocation-positive and 1/3 of ETV6 translocation-negative secretory carcinomas). Pathogenic variants of *MLH1, MUTYH*, and *STK11* were also identified. Variants of uncertain significance included mutations in *KMT5A*. These novel characteristic genetic alterations may advance current understandings of secretory carcinoma tumorigenesis and progression, leading to improved diagnoses and treatment strategies.

## Introduction

Secretory carcinoma is a recently described salivary gland neoplasm characterized by the chromosomal translocation t(12;15)(p13;q25), which results in the *ETV6-NTRK3* fusion gene. This gene is identical to the one found in secretory carcinoma of the breast, infantile fibrosarcoma, and acute myeloid leukemia [1–3].

Since the description of secretory carcinoma as a new disease entity, several studies have reported the clinicopathologic features of secretory carcinoma that allow for its differential diagnosis from other subtypes of salivary gland tumors, such as acinic cell carcinoma, adenocarcinoma—not otherwise specified, low-grade mucoepidermoid carcinoma, and intraductal carcinoma, or so-called low-grade cribriform cystadenocarcinoma. The histologic features of secretory carcinoma include a variety of architectural growth patterns, the presence of intracytoplasmic vacuoles, mucin-like and colloid-like eosinophilic secretions, and a lack of intracytoplasmic periodic acid-Schiff^+^ (PAS^+^) zymogen granules. Immunohistochemistry analyses have shown that most secretory carcinomas are positive for S100 and mammaglobin and negative for DOG1 [2, 4–15]. Secretory carcinoma reveals relatively frequent rates of lymph node metastasis in as many as 25% of cases at presentation [2]. This tumor usually shows indolent clinical behaviors; however, aggressive courses, such as recurrence and death of disease, are also reported in some cases [1, 2, 4, 16–18].

Apart from the *ETV6* gene translocation, little is known about genomic alterations in secretory carcinoma. Therefore, further studies of the novel recurrent genetic alterations in secretory carcinoma are needed. In this study, we characterized the genetic alterations in secretory carcinoma through targeted deep sequencing analyses to detect novel recurrent somatic mutations.

## Materials and methods

### Case selection

The database of Severance Hospital Cancer Registry Data (Seoul, South Korea) was searched to identify secretory carcinoma candidates from salivary gland tumors originally diagnosed as acinic cell carcinoma, adenocarcinoma—not otherwise specified, various subtypes of adenocarcinoma, and mucoepidermoid carcinoma. Patients with other malignancies were not included. The tumors were from patients treated and monitored at the Yonsei University Health System Department of Otorhinolaryngology. Patient medical records, pathology reports, and clinical details were reviewed in accordance with the Eighth American Joint Committee on Cancer criteria [19]. The study was approved by the Severance Hospital Institutional Review Board (Protocol No. 4-2018-0816). Supplementary Fig. 1 shows a flowchart and details of the study.

After an initial screening of histology and clinical data, all cases of acinic cell carcinoma and adenocarcinoma not otherwise specified in salivary glands were selected for further screening. Two independent pathologists (KN and SOY) reviewed all available stained slides by routine light microscopy and selected the most representative formalin-fixed paraffin-embedded block for ancillary tests. Histologic classification followed the World Health Organization classification criteria [2]. Candidate cases were selected for *ETV6* gene rearrangement fluorescence in situ hybridization (FISH) analyses on the basis of having low-grade nuclear features with bubbly eosinophilic cytoplasm, no zymogen granules, a positive staining for S100 and/or mammaglobin, and a negative staining for DOG1 and P63. Twenty two of the reviewed cases were considered to be secretory carcinoma. Among them, 19 secretory carcinomas harbored *ETV6* translocation and 3 secretory carcinomas lacked *ETV6* translocation. These 3 *ETV6* translocation-negative cases, which were originally diagnosed as acinic cell carcinoma, had a histology similar to that of secretory carcinoma in that they showed microcystic, tubular, and papillary cystic architectures, low-grade uniform nuclei, occasional small nucleoli, pinkish bubbly cytoplasm, lack of PAS+zymogen granules, and mucin-like and colloid-like eosinophilic secretions. They also displayed diffuse positive staining for both S100 and mammaglobin and negative staining for DOG1 and P63. These cases did not demonstrate features favoring low-grade mucoepidermoid carcinoma, such as squamoid differentiation or intracytoplasmic mucin. Also, the lack of micropapillary or sieve-like fenestrated architectures and the presence of invasive foci were different from typical histological features of low-grade salivary duct carcinoma [2]. Given their present histological features and immunophenotypes typical of secretory carcinoma, these 3 cases were included as secretory carcinoma as suggested [20].

During the review process, we also reviewed 36 acinic cell carcinoma cases. To further identify the characteristics of secretory carcinoma that distinguish it as a clinicopathologically distinct disease entity, we compared the clinical and histologic features of 22 secretory carcinomas with those of the 36 cases of acinic cell carcinoma.

### Tissue microarray preparation and immunohistochemistry

To construct the tissue microarray, two different representative tumor areas per sample were selected, and 3 mm tissue cores were taken from formalin-fixed paraffin-embedded donor tissue blocks and arranged in recipient tissue microarray blocks using a trephine apparatus. A Ventana Benchmark XT autostainer (Ventana Medical Systems, Inc, Tucson, AZ, USA) was used to perform immunohistochemistry analyses for mammaglobin (clone 31A5, 1:100; Cell Marque, Rocklin, CA, USA), S100 (clone Z0311, 1:2000; Dako, Glostrup, Denmark), DOG1 (clone SP31, Ready-to-Use; Cell Marque), p63 (clone M7317, 1:200; Dako), and androgen receptor (RTU, clone SP107; Cell Marque). The immunohistochemistry analyses were performed on 4 µm thick sections prepared from the tissue microarray tissue blocks and representative formalin-fixed paraffin-embedded tissues when necessary, in accordance with standard protocols [21].

### Fluorescence in situ hybridization

FISH was performed on representative formalin-fixed paraffin-embedded tissue sections using a commercially available *ETV6* dual-color break-apart probe (07j77-001; Abbott Molecular, Des Plaines, IL, USA), as previously described [22]. Cells with the *ETV6* rearrangements had split orange and green signals that were apart from each other. Tumors were considered to be positive for *ETV6* if this rearrangement was found in >15% of cells.

### Targeted deep sequencing analyses

All targeted deep sequencing analyses (including related experiments and genome analyses) were performed at Macrogen (Seoul, South Korea). A customized panel (Axen Cancer Master Panel, Macrogen) including 535 genes for SNV/InDel, 54 genes for fusions, and 1 promoter gene was designed and used for the targeted sequencing (See Supplementary Table 1 for the entire list of genes) of 20 secretory carcinoma samples (17 *ETV6* translocation-positive and 3 *ETV6* translocation-negative secretory carcinomas). Paired-end sequencing (2 × 150 bp) was performed using a NextSeq 500 sequencer (Illumina, San Diego, CA, USA). Genome analyses were performed to detect somatic short variants following GATK (Genome Analysis Toolkit) Best Practices [23]. The adapter sequences were removed using Cutadapt [24]. The raw sequence reads in FASTQ files were mapped to the UCSC hg19 human reference by using BWA-mem [25]. Poorly mapped reads with mapping quality values <20 were removed using Samtools [26]. Picard MarkDuplicates (http://broadinstitute.github.io/picard) were used to exclude PCR artificial duplicates. Indel realignment and base quality score recalibration were performed using GATK, and the Mutect2 algorithm in the GATK was used to call somatic single nucleotide variants and indels [27]. False positive variant calls originating from oxoG artifacts were excluded. In addition, mutations with a variant allele frequency <3% and a 100× total depth were excluded. Germline variants were excluded when the minor allele frequency was ≥5% in the Exome Aggregation Consortium (http://exac.broadinstitute.org/) or the Macrogen Korean Population Database [28]. The functional impacts of the variants were determined using SnpEff v4.3 and SnpSift with dbNSFP v2.9.3 [29–31]. Variants were further filtered using the following cut-off values: mutant allele count <5 and an allele frequency in the normal population (from dbSNP) >0.1 [32]. All remaining variants were manually inspected by sequence analysis experts using the Integrative Genomics Viewer [33]. The fusion genes were analyzed using an in-house script (Macrogen) that was able to discriminate plausible gene fusion events among the structural variations predicted by LUMPY [34] and UMI Error Correction Local App 1.0.0.1 (Illumia; https://support.illumina.com/downloads/umi-error-correction-local-app-documentation.html). Copy number variations were analyzed using an in-house script (Macrogen). The clinical significance of genetic variants was determined on the basis of the ClinVar variation report (https://www.ncbi.nlm.nih.gov/clinvar).

### Statistical analyses of patient survival

The Kaplan–Meier method was used to analyze survival rates, and differences were compared using the log-rank test. Overall survival was measured from the date of diagnosis to the date of death or last follow-up. Disease-free survival was measured from the date of diagnosis to cancer recurrence, continuance of stable disease/partial remission/progressive disease without complete remission, or cancer-related death during the study period. Two-sided *P* values < 0.05 were considered statistically significant. Statistical analyses were conducted using IBM SPSS 23 software for Windows (IBM Corp, Armonk, NY, USA).

## Results

### Histological features and clinical outcomes of secretory carcinoma

**Fig. 1:**
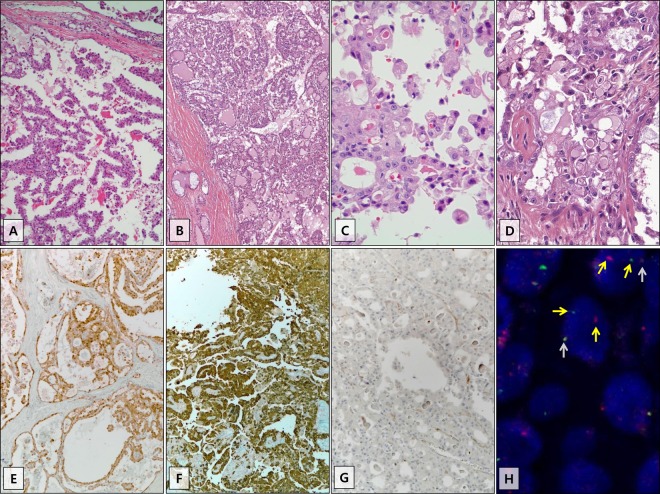
Histopathological features of secretory carcinoma. Tumors frequently show papillary cystic (**a**) and microcystic (**b**) growth architectures. The tumor cells show low-grade uniform nuclei, occasional small nucleoli, and pinkish bubbly cytoplasm (**c**). Mucin-like and/or eosinophilic secretions are easily detected in tumors with microcystic growth patterns (**b**, **d**). The tumors showed expression of S100 (**e**) and mammaglobin (**f**) and no expression of DOG1 (**g**). An *ETV6* break-apart FISH assay showed a split of one red signal and one green signal per nucleus, indicative of *ETV6* translocation (H: yellow arrow) with a fusion signal (H: gray arrow). The images in A through G were captured at ×100 and ×400 magnification.

**Fig. 2:**
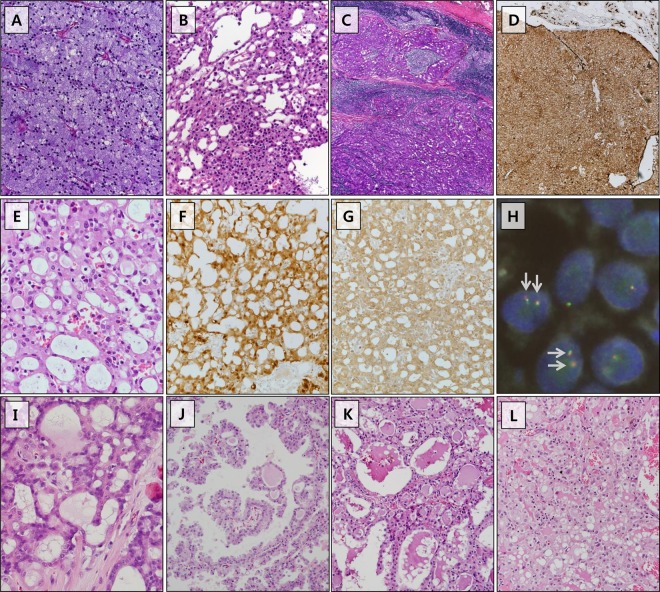
Histologic features of acinic cell carcinoma and *ETV6* translocation-negative secretory carcinoma. Features of typical acinic cell carcinoma or blue dot tumor (**a**). Microcystic acinic cell carcinoma showing focal zymogen granule-containing cells (**b**). The tumor stroma is sometimes lymphocyte predominant (**c**). Typical immunohistochemistry of acinic cell carcinoma showing DOG1 positivity (**d**). A case of *ETV6* translocation-negative secretory carcinoma showing microcystic architecture without definite zymogen granules (E) and diffuse staining for S100 (**f**) and mammaglobin (**g**). The tumor shows an intact *ETV6* gene by FISH. Most tumor nuclei show intact fusions (**h**, red and green signals; gray arrows). This case showed *STK11* gene mutation. Another case of *ETV6* translocation-negative secretory carcinoma showing microcystic architecture (**i**). This case revealed no remarkable genetic alteration. Another *ETV6* translocation-negative secretory carcinoma showing variegated architectures of predominantly papillary cystic, partly microcystic architecture, and focally solid growth patterns (**j**–**l**). This case showed *PRSS1* mutation. The tumor cells of those three *ETV6* translocation-negative secretory carcinomas showed low-grade uniform nuclei, occasional small nucleoli, and pinkish bubbly cytoplasm (**e**, **i**–**k**). Mucin-like and/or eosinophilic secretions are easily detected in tumors with microcystic growth patterns (**e**, **i**, **k**). Further features of the three cases of *ETV6* translocation-negative secretory carcinoma are summarized in Supplementary Fig. 3.

The histopathologic and clinical features of the 22 secretory carcinoma cases are represented in Figs. 1 and 2 and summarized in Tables 1 and 2. The characteristics of the 36 acinic cell carcinoma cases are summarized for comparison and represented in Fig. 2 and Tables 1 and 2. Details are described in the Supplementary Appendix and Supplementary Figs 2 and 3.

**Table 1:** Histologic features of secretory carcinoma.

Variables	Secretory carcinoma (*N* = 22)	Acinic cell carcinoma (*N* = 36)
Gross appearance		
Single-round	15 (68%)	16 (44%)
Multi-lobulated	7 (32%)	20 (56%)
Tumor border		
Pushing	14 (64%)	25 (69%)
Infiltrative	8 (36%)	11 (31%)
Dominant growth pattern		
Solid	0 (0%)	29 (81%)
Microcystic	6 (27%)	7 (19%)
Papillary cystic	14 (64%)	0 (0%)
Cribrifrom	2 (9%)	0 (0%)
Tumor stroma		
Sclerosis	22 (100%)	31 (86%)
Lymphocyte-rich	0 (0%)	5 (14%)
Zymogen granule		
Prominent	0 (0%)	30 (83%)
Focally present	0 (0%)	6 (17%)
Absent	22 (100%)	0 (0%)
Lymphovascular invasion	0 (0%)	2 (6%)
Perineural invasion	2 (9%)	4 (11%)
Immunohistochemistry		
DOG1 expression	2 (9%; focal)	36 (100%; 36 diffuse)
S100 expression	22 (100%; 21 diffuse and 1 focal)	0 (0%)
Mammaglobin expression	20 (91%; 17 diffuse and 3 focal)	0 (0%)
P63 expression	0 (0%)	0 (0%)
*ETV6* gene		
Translocated	19 (86%)	Not done
Not translocated	3 (14%)

**Table 2:** Clinicopathologic features and clinical outcomes of secretory carcinoma.

Variables	Secretory carcinoma (*N* = 22)	Acinic cell carcinoma (*N* = 36)
Median age (year)	34 (mean, 36.5; range, 6–78)	34 (mean, 37.9; range, 14–68)
Male: Female	14:8	14:22
Anatomical distribution		
Parotid gland	20 (91%)^a^	35 (97%)
Minor salivary gland	1 (5%)^a^	1 (3%)
Submandibular gland	1 (5%)^a^	0
Median tumor size (cm)	2.2 (mean, 2.3; range: 1.1–3.8)	2.7 (mean, 2.5; range, 1.0–5.2)
Surgery		
Superficial parotidectomy	13 (59%)	22 (61%)
Total parotidectomy	7 (32%)	13 (36%)
Mass excision	2 (9%)	1 (3%)
Lymph-node dissection		
Performed	11 (50%)	5 (14%)
Not performed	11 (50%)	31 (86%)
Nodal metastasis^b^	4 out of 11	0 out of 5
Resection margin involvement	2 (9%)	3 (8%)
American Joint Committee on Cancer tumor stage		
I (T1N0)	3 (14%)^c^	12 (33%)^b^
II (T2N0)	14 (64%)^c^	21 (58%)^b^
III (T3 or N1)	5 (23%)^c^	3 (8%)^b^
Post operative radiation therapy	8 (36%)	5 (14%)
Median follow-up period (mo)	46 (mean, 53.8; range, 6–140)	48 (mean, 58.5; range, 6–180)
Overall recurrence	7 (32%)	5 (14%)
Recurrence site		
Local	5	3
Nodal	1	0
Local and nodal	1	2
Median recurrence interval (mo)	24 (mean, 24.5; range: 6–51)	48 (mean, 46; range: 12–96)
Mean disease-free survival (mo)	99 (95% CI, 74–123)	153 (95% CI, 128–177)
3-year recurrence rate^e^	5/18 (28%)	2/26 (8%)
5-year recurrence rate^e^	7/16 (44%)	4/17 (24%)
Died of disease	0 (0%)	0 (0%)

^a^The percentages before rounding are 90.9%, 4.5%, and 4.5%, respectively

^b^Lymph-node metastasis was known only among cases undergoing lymph node dissection. To avoid overestimation, the percentage was not presented in this table

^c^The percentages before rounding are 13.6%, 63.6%, and 22.7%, respectively. In 11 patients without node dissection, clinical N stage was considered as cN0

^d^The percentages before rounding are 33.33%, 58.33%, and 8.33%, respectively

^e^Three-year and five-year follow-up data were available for 18 and 16 patients with secretory carcinoma, respectively; and 26 and 17 patients with acinic cell carcinoma, respectively

Histologically, papillary cystic growth was the most common pattern observed in the secretory carcinoma cases (64% [14/22]). The tumor cells showed low-grade uniform nuclei, occasional small nucleoli, and pinkish bubbly cytoplasm. PAS^+^ zymogen granules were not observed, but mucin-like and colloid-like eosinophilic secretions were easily observed. All secretory carcinomas were diffusely stained for S100 in both the nuclei and cytoplasm, except for one case with focal expression. Mammaglobin expression presented in 91% (20/22) of the secretory carcinomas as a cytoplasmic staining pattern and showed diffuse expression in 77% (17/22). None of the secretory carcinomas showed diffuse expression of DOG1 or p63. The *ETV6* gene rearrangement was noted in 86% (19/22) in FISH analyses (Table 1). Cervical lymph node dissection was performed in 50% (11/22) of patients with secretory carcinoma; among them, four had nodal metastasis. The American Joint Committee on Cancer tumor stage distribution of our series was I (14% [3/22]), II (64% [14/22]), and III (23% [5/22]). The median follow-up for patients with secretory carcinoma was 46 months (range, 6-140 months). During the follow-up period, 32% (7/22) of patients experienced disease recurrence, including local (23% [5/22]), nodal (5% [1/22]), and local and nodal disease (5% [1/22]). The median interval period between surgery and recurrence was 24 months (range, 6–51 months). None of the patients died from disease during the follow-up period (Table 2). When comparing rates of disease-free survival between secretory carcinoma and acinic cell carcinoma patients, no statistical significance was observed (Supplementary Fig. 4).

### Status of gene alterations

**Fig. 3:**
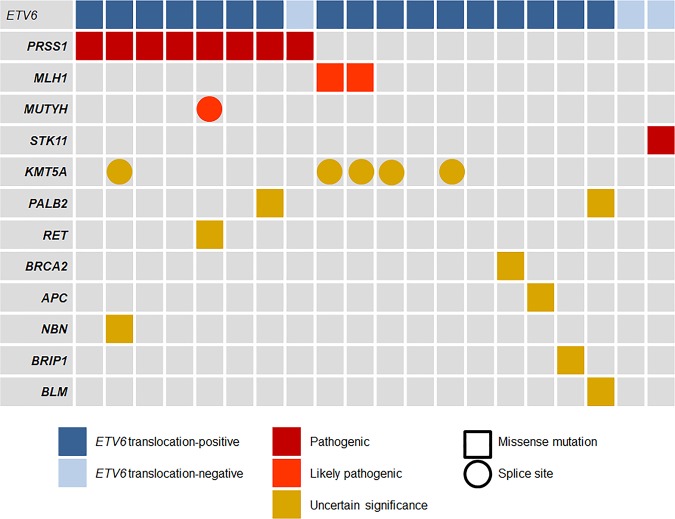
Landscape of the genetic alterations identified by the targeted deep sequencing analysis for 20 secretory carcinomas.

Twenty tumor tissue samples consisting of secretory carcinomas (17 *ETV6* translocation-positive and 3 *ETV6* translocation-negative secretory carcinomas) were subjected to targeted deep sequencing analyses. Genetic variants were selected for screening if they were pathogenic, likely pathogenic, of uncertain significance in the ClinVar variation report (https://www.ncbi.nlm.nih.gov/clinvar), and among those observed in human cancers (Fig. 3 and Supplementary Table 2).

In our series, four genes were identified as pathogenic or likely pathogenic. These included missense mutations and splice site mutations in *PRSS1* (c.47C>T; p.A16V), *MLH1* (c.1151T>A; p.V384D), *MUTYH (*c.934-2A>G; splice site), and *STK11 (*c.842C>T; p.P281L). The most commonly detected pathogenic variant was the mutation in the *PRSS1* gene, which was detected in 40% (8/20; 7/17 of *ETV6* translocation-positive and 1/3 of *ETV6* translocation-negative secretory carcinomas). Mutations in *MLH1* and *MUTYH* were noted in secretory carcinoma (*n* = 2 and 1, respectively), and serine/threonine kinase 11 (*STK11)*, a gene associated with the phosphatidylinositol 3-kinase (PI3K) pathway, was observed (*n* = 1) [35]. As a variant of uncertain significance, *KMT5A* (c.290-3C>A; splice site) mutations were recurrently observed in 25% (5/20) of secretory carcinomas. Although rare, several variants of uncertain significance were noted in secretory carcinoma; these were mutations in *PALB2* (c.2329G>A; p.D777N), *RET* (c.2611G>A; p.V871I), *BRCA2* (c.6029T>G; p.V2010G), *APC* (c.1276G>T; p.A426S), *NBN* (c.505C>T; p.R169C), and *BRIP1* (c.485G>A; p.R162Q) (Fig. 3). None showed a significant copy number alteration.

### Clinical implications of gene alterations

**Fig. 4:**
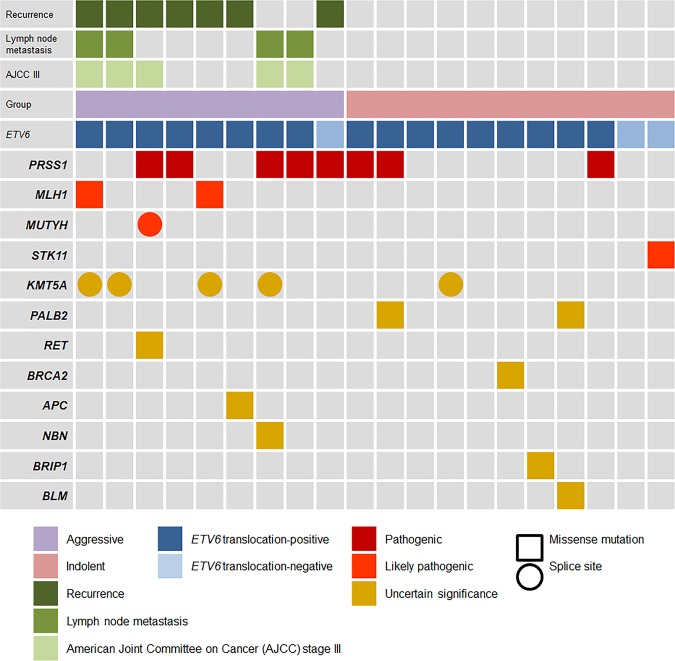
The genetic alteration status between the case group with aggressive clinicopathologic features, such as tumor recurrence, lymph node, and/or advanced American Joint Committee on Cancer stage III, and the case group of indolent features showing no evidence of tumor recurrence, lymph node metastasis, or advanced American Joint Committee on Cancer stage III.

The tested cases were divided into two groups. The cases were classified into the aggressive group (9 secretory carcinomas) if they had at least one of following features: the presence of lymph node metastasis, were classified as American Joint Committee on Cancer stage III, and/or had tumor recurrence during the observation period. The cases classified into the indolent group (11 secretory carcinomas) did not include any of the above-mentioned features. The genetic alterations analyzed in each group are represented in Fig. 4.

The mutation frequency of PRSS1 was 56% (5/9) in the aggressive group and 27% (3/11) in the indolent group. Mutations in MLH1 and MUTYH genes are involved in DNA repair and the development of cancers, especially colorectal cancer (ClinVar; https://www.ncbi.nlm.nih.gov/clinvar/) [36]. Mutations in these genes were only observed in the aggressive group. The KMT5A gene is involved in histone modification due to its function as a histone methyltransferase [37] (ClinVar; https://www.ncbi.nlm.nih.gov/clinvar/). Although a variant of uncertain significance, KMT5A was found to have a mutation rate of 44% (4/9) in the aggressive group and 9% (1/11) in the indolent group.

### Results of RNA fusion gene analysis

The RNA extracted from the formalin-fixed paraffin-embedded tissues was severely degraded, and RNA quality was very low. Nevertheless, RNA fusion gene analyses detected several gene fusion types. The fusion variant of *ETV6- NTRK3* was noted in 4 out of 17 *ETV6* translocation-positive secretory carcinomas, which were defined by FISH assays. This fusion variant was not detected in any of the 3 *ETV6* translocation-negative secretory carcinomas. Single-event fusion variants were identified (including *ETV6* with *NTRK2, PAX5*, or *ABL1* and fusion scripts of *LPP- KMT2A, QKI- NTRK2, NUP214- ABL1, and BRAF- MKRN1)* with a read depth below 5. Details are summarized in Supplementary Table 3.

## Discussion

We characterized the novel recurrent somatic mutations in secretory carcinoma using targeted deep sequencing analyses. To our knowledge, no recurrent genomic alterations in secretory carcinoma, except for the *ETV6* rearrangement, have been reported.

In our screening for secretory carcinoma candidates, we noted that zymogen granule-poor tumors that were initially diagnosed as acinic cell carcinoma constituted the largest source of secretory carcinomas, as reported in previous studies [4–14]. Although secretory carcinoma is the disease entity most commonly mimicked by acinic cell carcinoma [2, 38], we confirmed that secretory carcinoma had several distinct features. Clinically, patients with secretory carcinoma had relatively frequent lymph node metastasis. In our series, at least 4 out of 22 (18.2%) secretory carcinomas were pathologically proven to be lymph node-positive. The rates of lymph node metastasis have been reported in as many as 25% cases in literatures [2]. A previous report shows about 22% (4/18) of lymph node metastases among patients with secretory carcinoma. Although statistical significance was not determined when combining additional cases with known nodal status from previously reported cases, the proportion of secretory carcinoma cases with nodal metastasis (17.6%, 6/34) remained slightly higher than acinic cell carcinoma (3/38, 7.9%) [4, 16]. These findings suggest the need for greater attention to neck lymph node metastasis in the treatment of patients with secretory carcinoma, including the surgical evaluation of lymph nodes. Furthermore, the aggressive clinical course in some secretory carcinoma cases like as tumor recurrence as shown in our series and other literatures [1, 2, 4, 16–18] suggests that secretory carcinoma may require risk stratification for patient treatment, despite the possibility of secretory carcinoma being a low-grade salivary gland carcinoma like acinic cell carcinoma. Histologically, we found the differential characteristics of secretory carcinoma to be the papillary cystic growth pattern, the lack of PAS^+^ zymogen granules, and the distinct immunohistochemical profiles of S100 and/or mammaglobin positivity as well as DOG1 negativity, as previously described [2, 4–15, 21, 38].

The comprehensive targeted deep sequencing analyses of the secretory carcinoma cases were performed using cancer-related gene panels. These analyses revealed novel recurrent somatic mutations, especially in the *PRSS1* gene. Furthermore, our analyses provided biologic insight into genetic alterations of secretory carcinoma.

The *PRSS1* gene encodes a cationic trypsinogen, which is a member of the trypsin family of serine proteases. This enzyme, which aids in digestion, is secreted by the pancreas and cleaved to its active form in the small intestine. Germline and somatic mutations in the *PRSS1* gene are associated with hereditary pancreatitis, chronic pancreatitis, and pancreatic adenocarcinoma. As a result of *PRSS1* gene mutations, trypsinogen is prematurely activated and cannot be broken down because of the elimination of the trypsin hydrolysis site. The resulting increase in trypsin is thought to be associated with pancreatitis and pancreatic adenocarcinoma. The R117H mutation is a frequently observed germline mutation in *PRSS1*. N29I, A16V, D22G, K23R, A121T, and R122C mutations in the *PRSS1* gene have also been reported to be rare mutations in cationic trypsinogen [39–43]. Among these mutations, we recurrently observed the A16V(C→T) mutation in 40% (8/20) of secretory carcinoma cases. It may be the first identification of this mutation in secretory carcinoma.

Pancreatic digestive enzymes, including trypsin, are also expressed in salivary glands. Because both the pancreas and salivary glands are exocrine glands with a very similar histology [44, 45], the *PRSS1* mutation and resulting abnormal trypsinogen function may also be involved in the pathogenesis of salivary gland cancer. The identification of a recurrent A16V mutation in the *PRSS1* gene in secretory carcinoma is of clinical interest and warrants further in-depth studies as well in other subtypes of salivary gland tumors.

Secretory carcinoma is most commonly reclassified from zymogen granule-poor acinic cell carcinoma. Given that zymogen granules contain many digestive enzymes, such as trypsin, mutant *PRSS1* may be involved in the tumorigenesis of this zymogen granule–poor cancer. *ETV6-NTRK3* fusion may be associated with a secondary mutation in *PRSS1*, as secretory carcinoma is characterized by *ETV6* gene rearrangement, with its most common translocation partner being the *NTRK3* gene. There have been no studies on the relationship between *PRSS1*, *ETV6* (a transcription repressor), and *NTRK3* (a neurotropic tyrosine kinase). However, in our series, 1 case of SC with an intact *ETV6* gene had the *PRSS1* mutation, suggesting that other mechanisms may be involved in the *PRSS1* mutation of zymogen granule-poor cancers.

Genetic profiling showed that the secretory carcinomas with intact *ETV6* genes had genetic alterations that were not significantly different from those of the secretory carcinomas with *ETV6* gene translocation. In addition, the *PRSS1* mutation was shared by both. Therefore, the typical histology of secretory carcinoma, including the papillary cystic and microcystic growth pattern, the lack of PAS^+^ zymogen granules, and the distinct immunohistochemical profiles of S100 and/or mammaglobin positivity [2, 4–15, 21, 38] supported the diagnosis of secretory carcinoma.

The 3 *ETV6* translocation-negative secretory carcinomas originally diagnosed as zymogen granule-poor acinic cell carcinoma posed a diagnostic dilemma. The International Head and Neck Scientific Group recently proposed classifying these tumors as a family of tumors rather than distinct entities, such as carcinomas of intercalated duct-like cells in microcystic, papillary follicular, cystic, and mixed architectural arrangement among the acinic-intercalated ductal carcinoma [38]. In our series, however, we followed the current suggestion [20] and grouped the 3 *ETV6* translocation-negative cases as secretory carcinoma.

Detection of *ETV6* by FISH is technically feasible and has been described in most cases of secretory carcinoma since its original description. However, there were several reports showing that FISH did not detect *ETV6* translocation in 5 to 14% of tested cases with typical histological features of secretory carcinoma [10, 17, 20]. In our series, 14% (3/22) of secretory carcinomas failed to detect *ETV6* translocation by FISH analyses, and RNA fusion gene analyses also did not detect *ETV6* gene fusion in those 3 cases. The negative results of *ETV6* translocation by FISH analysis may stem from technical limits.

Secretory carcinoma did not display highly diverse or highly frequent pathogenic variants that are known to be cancer genomes in highly malignant human cancers [46]. Rather, the low-grade, indolent behaviors of secretory carcinoma were observed. Nevertheless, cases with aggressive clinicopathologic features, such as lymph node metastasis, advanced American Joint Committee on Cancer stage, and tumor recurrence, revealed some gene alterations of *PRSS1, MLH1, MUTYH*, and *KMT5A*. These gene alterations may help to classify the high risk group among patients diagnosed with secretory carcinoma.

A recent study has shown alternative *ETV6* partners, such as *RET* and *MAML3*, by using the targeted next-generation sequencing assay [47]. Other studies have shown *ETV6-RET* fusion by using reverse transcription polymerase chain reaction [48]. Due to the low quality of RNA in our series, we could not reliably identify fusion scripts. We only observed the *ETV6-NTRK3* fusion and the prototypical fusion in secretory carcinoma [1, 2]. The fusion was observed in 4 of *ETV6* translocation-positive secretory carcinoma, but not any of *ETV6* translocation-negative secretory carcinomas. As alternative fusion partners, *NTRK2*, *ABL1*, and *PAX5* were also identified, but the read depth was too low and unreliable.

In addition to the *ETV6* gene rearrangement, the A16V mutation in *PRSS1* was recurrently observed in secretory carcinoma, which are generally low-grade indolent tumors. However, cases with aggressive clinicopathologic features have some recurrent gene alterations in *PRSS1*, *MLH1, MUTYH*, and *KMT5A*. Regarding their distinct clinical and histological features, including relatively frequent rates of lymph node metastasis and tumor recurrence, these novel genetic alterations may provide insight into the biological pathogenesis of secretory carcinoma. They may also help to improve current diagnostic methods, patient treatment strategies, such as lymph node evaluation and follow-up for tumor recurrence, clinical risk stratification of patients, and therapeutic targets for recurrent intractable tumors.

## Supplementary information

Supplementary Appendix

Supplementary Figure 1

Supplementary Figure 2A

Supplementary Figure 2B

Supplementary Figure 2C

Supplementary Figure 2D

Supplementary Figure 3A

Supplementary Figure 3B

Supplementary Figure 3C

Supplementary Figure 4

Supplementary Table 1

Supplementary Table 2

Supplementary Table 3
