# Supplementary material for: Characterization of novel genetic alterations in salivary gland secretory carcinoma
Source: Mod Pathol. 2019 Dec 10;33(4):541–50. doi: 10.1038/s41379-019-0427-1 (PMC7113190; doi:10.1038/s41379-019-0427-1)
Supplement: Supplementary file 1 — Supplementary Appendix [file 41379_2019_427_MOESM1_ESM.docx]

**Supplementary appendix**

**Characterization of novel genetic alterations of salivary gland secretory**

**carcinoma**

Kiyong Na^1,2^, Juan Carlos Hernandez-Prera^3^, Jae-Yol Lim^4^, Ha Young Woo^1^, and Sun Och Yoon^1, 3^

^1^Department of Pathology, Yonsei University College of Medicine, Severance Hospital,

Seoul, South Korea

^2^Department of Pathology, College of Medicine, Kyung Hee University, Seoul, South Korea

^3^Department of Anatomic Pathology, H. Lee Moffitt Cancer Center and Research Institute,

Tampa, FL, USA

^4^Department of Otorhinolaryngology, Yonsei University College of Medicine, Seoul, South

Korea

**Contents**

Results

Legends for Supplementary Figures and Supplementary Tables

**Histopathologic features of secretory carcinoma**

The histopathologic features of the 22 cases of secretory carcinoma are summarized in Table 1. The characteristics of the 36 acinic cell carcinoma cases, which is the top differential entity from secretory carcinoma are also summarized for comparison. The representative pictures are displayed in Figure 1 and 2 and Supplementary Figures 2 and 3.

Most cases of secretory carcinoma (68%, 15/22) presented as a single-round mass, whereas multilobulated masses were noted in 32% (7/22) of secretory carcinoma cases. Most (64%, 14/22) secretory carcinomas were well circumscribed and were characterized by multiple macrocysts or unicysts with intracystic proliferation. Diffuse infiltration into the surrounding tissue was observed in 36% (8/22) of cases. Histologically, secretory carcinomas showed varying proportions of solid, microcystic, papillary cystic, and cribriform growth patterns. Regarding the dominant growth pattern in each case, predominantly papillary cystic growth was the most common pattern (64%, 14/22), followed by predominantly microcystic and predominantly cribriform patterns. A dominantly solid growth pattern was not observed in any case. The stroma of the secretory carcinomas was associated with various

degrees of sclerosis, hemorrhage, hemosiderin, and cholesterol clefts. However, lymphocyte-rich stroma was not observed.

Cytologically, the tumor cells showed low-grade uniform nuclei, occasional small

nucleoli, and pinkish, bubbly cytoplasm. PAS-positive zymogen granules were not observed

in any secretory carcinoma. Mucin-like, colloid-like eosinophilic secretions were observed and were easily detected in tumors with microcystic or cribriform growth patterns, although some cases did not have these secretions. Squamoid differentiation was not present. All secretory carcinomas were diffusely stained for S100 in both the nuclei and cytoplasm, except for one case with focal expression. Mammaglobin expression was present in 91% (20/22) of the secretory carcinomas as a cytoplasmic staining pattern, showing diffuse expression in 77% (17/22) of the secretory carcinomas. None of the secretory carcinomas showed diffuse expression of DOG1 or p63. *ETV6* gene arrangement was detected in 86% (19/22) by FISH analyses (Table 1).

**Clinical features and outcomes of secretory carcinoma**

The clinicopathologic features and clinical outcomes for the 22 cases of secretory carcinoma are summarized in Table 2. The characteristics of the 36 cases of acinic cell carcinoma are also summarized for comparison.

For the 22 cases of secretory carcinoma, the median age at diagnosis was 34 years, and there was a male predilection (male to female ratio, 14:8). The majority of tumors (91%, 20/22) developed in the parotid gland, except for two cases that developed in the minor salivary and submandibular glands. The median tumor size was 2.2 cm (range, 1.1–3.8 cm). All tumors were treated with surgical resection. Cervical lymph node dissection was performed in 50% (11/22) of patients, and 4 of these patients had nodal metastasis. The American Joint Committee on Cancer tumor stage distribution was as follows: I, 14% (3/22); II, 64% (14/22); and III, 23% (5/22). Adjuvant radiation therapy was performed in 36% (8/22) of patients (Table 2).

The median follow-up for patients with secretory carcinoma was 46 months (range, 6–140 months). During the follow-up period, 32% (7/22) of patients experienced disease recurrence, including local (23%, 5/22), nodal (5%, 1/22), and local and nodal disease (5%, 1/22). The median interval between surgery and recurrence was 24 months (range, 6–51 months). Three-year and five-year follow-up data were available in 18 and 16 patients, respectively. In these patients, the 3-year and 5-year recurrence rates were 28% (5/18) and 44% (7/16), respectively. None of the patients died of disease during the follow-up period (Table 2).

**Histopathologic features of acinic cell carcinoma**

The acinic cell carcinomas (see Table 1) presented nearly equally as the multi-lobulated (56%, 20/36) and single-round (44%, 16/36) forms. A variable degree of infiltration into surrounding tissues was observed in 31% (11/36) of acinic cell carcinomas, while the

remaining 69% (25/36) of acinic cell carcinomas were well circumscribed. Acinic cell carcinomas demonstrated varying solid and microcystic growth patterns and a focal tubular architecture. Regarding the dominant growth pattern in each case, a predominantly solid pattern was the most common (81%, 29/36), followed by a predominantly microcystic growth pattern (19%, 7/36), and predominantly papillary cystic growth was not observed in acinic cell carcinomas. The solid growth pattern was characterized by sheets of cells separated by thin fibrovascular strands. The microcystic pattern was characterized by small cystic lumina in a loose aggregation of tumor cells. In most cases (86%, 31/36), stromal sclerosis was present in the tumor cell lobules to varying degrees (5–50% of the tumor area), but a minority (14%, 5/36) of cases showed lymphocyterich stroma with or without germinal centers. Hemorrhage, hemosiderin, and cholesterol clefts were not common features.

Cytologically, the tumor cells of acinic cell carcinomas showed low-grade uniform nuclei and occasional small nucleoli. Cytoplasmic vacuolization was observed in a minority of the tumor cells. Most (83%, 30/36) cases were zymogen granule-rich “classic” acinic cell carcinomas, which are also known as “blue dot tumors.” All of these classic acinic cell carcinomas showed diffuse DOG1 staining in a cytoplasmic and membranous pattern and no staining for S-100, mammaglobin, and p63.

Among the nine cases of zymogen granule-poor acinic cell carcinomas, six (17%, 6/36) showed pinkish cytoplasm with focal zymogen granules but immunohistochemical expression of DOG1, mammaglobin, S100, and P63, which was compatible with classic acinic cell carcinoma.

**Clinical features and outcomes of acinic cell carcinoma**

For the 36 cases of acinic cell carcinoma (see Table 2), the median age at diagnosis was 34 years, and there was slight female predilection (male:female, 14:22). Most (97%, 35/36) of the tumors occurred in the parotid gland. The median tumor size was 2.6 cm. All tumors were surgically resected, and 14% (5/36) of the patients also were treated with cervical lymph node

dissection. None of the five patients had nodal metastasis. Regarding American Joint Committee on Cancer tumor stage, stage II (58%, 21/36) was the most common, followed by stage I (33%, 12/36) and stage III (8%, 3/36). After surgery, 14% (5/36) of the patients were treated with radiation therapy. The median follow-up for the patients with acinic cell carcinoma was 48 months (range, 6–180 months). During follow-up, 14% (5/36) of patients experienced disease recurrence, including local disease (8%, 3/36) and local and nodal disease (6%, 2/36). The median interval between surgery and recurrence was 48 months (range, 12–96 months). Three-year and five-year follow-up data were available for 26 and 17 patients, respectively. In these patients, the 3-year and 5-year recurrence rates were 8% (2/26) and 24% (4/17), respectively. None of the patients died of disease during follow-up (Table 2).

**Legends for Supplementary Figures and Supplementary Tables**

**Supplementary Figure 1. Flowchart for the study**.

Nine cases that had the *ETV6* rearrangement as shown by fluorescence in situ hybridization

(FISH) were originally diagnosed as secretory carcinoma. Five of the nine secretory carcinoma cases were previously described in another study from our institution (1).

In 68% (36/53) of tumors originally diagnosed as acinic cell carcinoma, we observed cytoplasmic zymogen granules and immunohistochemistry (IHC) that was typical of acinic cell carcinoma, such as DOG1 positivity, and the diagnosis was not changed. However, 5 of the 53 (9%) cases were revised as oncocytoma (1/53) and low-grade mucoepidermoid carcinoma (4/53). The remaining 12 tumors originally diagnosed as acinic cell carcinoma were candidates for secretory carcinoma based on the histology and immunohistochemistry results, i.e., S100 and mammaglobin positivity and DOG1 negativity. In 15 tumors originally diagnosed as adenocarcinoma-not otherwise specified, 14 (93%) cases were reclassified as adenocarcinoma-not otherwise specified, salivary duct carcinoma, and high-grade mucoepidermoid carcinoma; and one case was a candidate for secretory carcinoma. Thirteen cases originally diagnosed as acinic cell carcinoma (n = 12) or adenocarcinoma-not otherwise specified (n = 1) were selected for FISH analysis. Of these 13 candidates, 10 were revised to secretory carcinoma because these cases had the *ETV6* translocation. The remaining three tumors were also classified as secretory carcinoma because they showed the histology and immunophenotype typical of secretory carcinoma although they had intact ETV6 gene status secretory carcinoma (2). For the targeted deep sequencing analysis, 22 secretory carcinomas were included. However, the nucleic acid extracted from the tumor tissue samples of two secretory carcinomas was not of sufficient quality for next generation sequencing analysis, and these tumors were excluded. Finally, 20 secretory carcinomas were subjected to targeted deep sequencing analysis.

**Supplementary Figure 2. Histologic features of acinic cell carcinoma**

Features of typical acinic cell carcinoma or blue dot tumor (A). Microcystic acinic cell carcinoma showing focal zymogen granule-containing cells (B). Sclerosis is variably noted in the tumor stroma, and the tumor stroma is sometimes lymphocyte predominant (C). Typical

immunohistochemistry of acinic cell carcinoma showing DOG1 positivity and mammaglobin and S100 negativity (D).

**Supplementary Figure 3. Histologic features of secretory carcinoma with intact *ETV6* gene**

Tumor showing a microcystic architecture without definite zymogen granules, and diffuse

staining for mammaglobin and S100, and negative staining for DOG1 (A). Another case of

*ETV6* translocation-negative secretory carcinoma showing microcystic architecture without

definite zymogen granules, diffuse staining for mammaglobin and S100, and negative

staining for DOG1 (B). Another *ETV6* translocation-negative secretory carcinoma showing

variegated architectures of predominantly papillary cystic, partly microcystic architecture,

and focally solid growth patterns, diffuse staining for S100 and mammaglobin, and negative

staining for DOG1(C). The tumor cells of those three ETV6 translocation-negative secretory

carcinoma show low-grade uniform nuclei, occasional small nucleoli, and pinkish bubbly

cytoplasm. Mucin-like and/or eosinophilic secretions are easily detected in tumors with

microcystic growth patterns.

**Supplementary Figure 4. Kaplan-Meier survival analysis for disease-free survival**

When comparing disease-free survival of secretory carcinoma patients to those of acinic cell carcinoma, no statistical significance was observed.

**Supplementary Table 1. Targeted Deep Sequencing Genes**

The Axen Cancer Master Panel (Macrogen, Seoul, South Korea) includes 535 genes for

SNV/InDel; 54 genes for fusions; and 1 promoter gene. All of the experiments, as well as the

genome analysis, for targeted deep sequencing were performed at Macrogen (Seoul, South

Korea).

**Supplementary Table 2. Mutation List of genes**

**Supplementary Table 3. RNA gene fusion analysis**

**References**

1. Woo HY, Choi EC, Yoon SO. Diagnostic Approaches for Salivary Gland Tumors with Secretory and Microcystic Features. Head Neck Pathol 2018;12:237-43.

2. Shah AA, Wenig BM, LeGallo RD, Mills SE, Stelow EB. Morphology in conjunction with

immunohistochemistry is sufficient for the diagnosis of mammary analogue secretory carcinoma. Head Neck Pathol 2015;9:85-95.
