# Supplementary figures and images for: Characterization of novel genetic alterations in salivary gland secretory carcinoma
Source: Mod Pathol. 2019 Dec 10;33(4):541–50. doi: 10.1038/s41379-019-0427-1 (PMC7113190; doi:10.1038/s41379-019-0427-1)

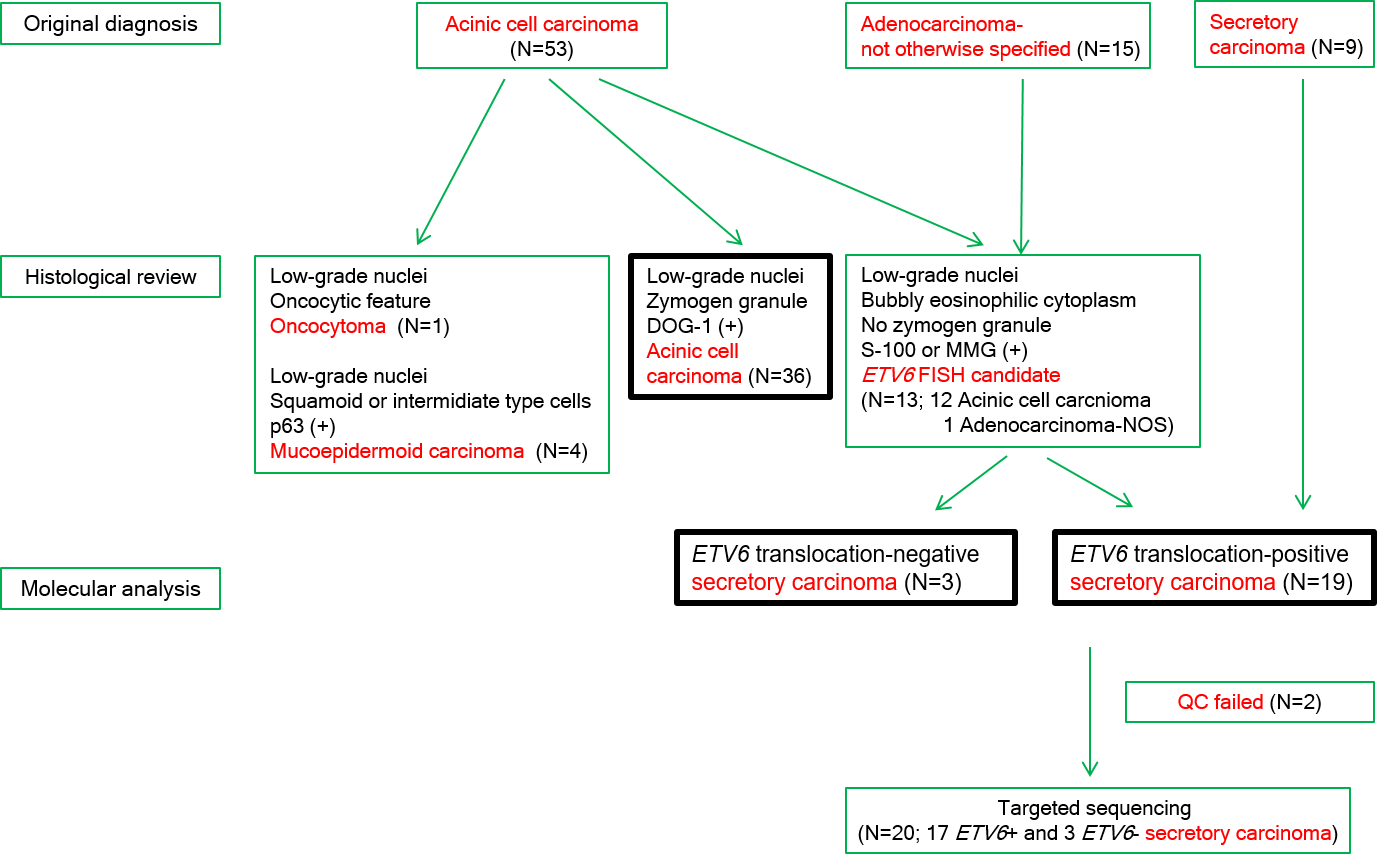

Supplement: Supplementary file 2 — Supplementary Figure 1 [file 41379_2019_427_MOESM2_ESM.tif]

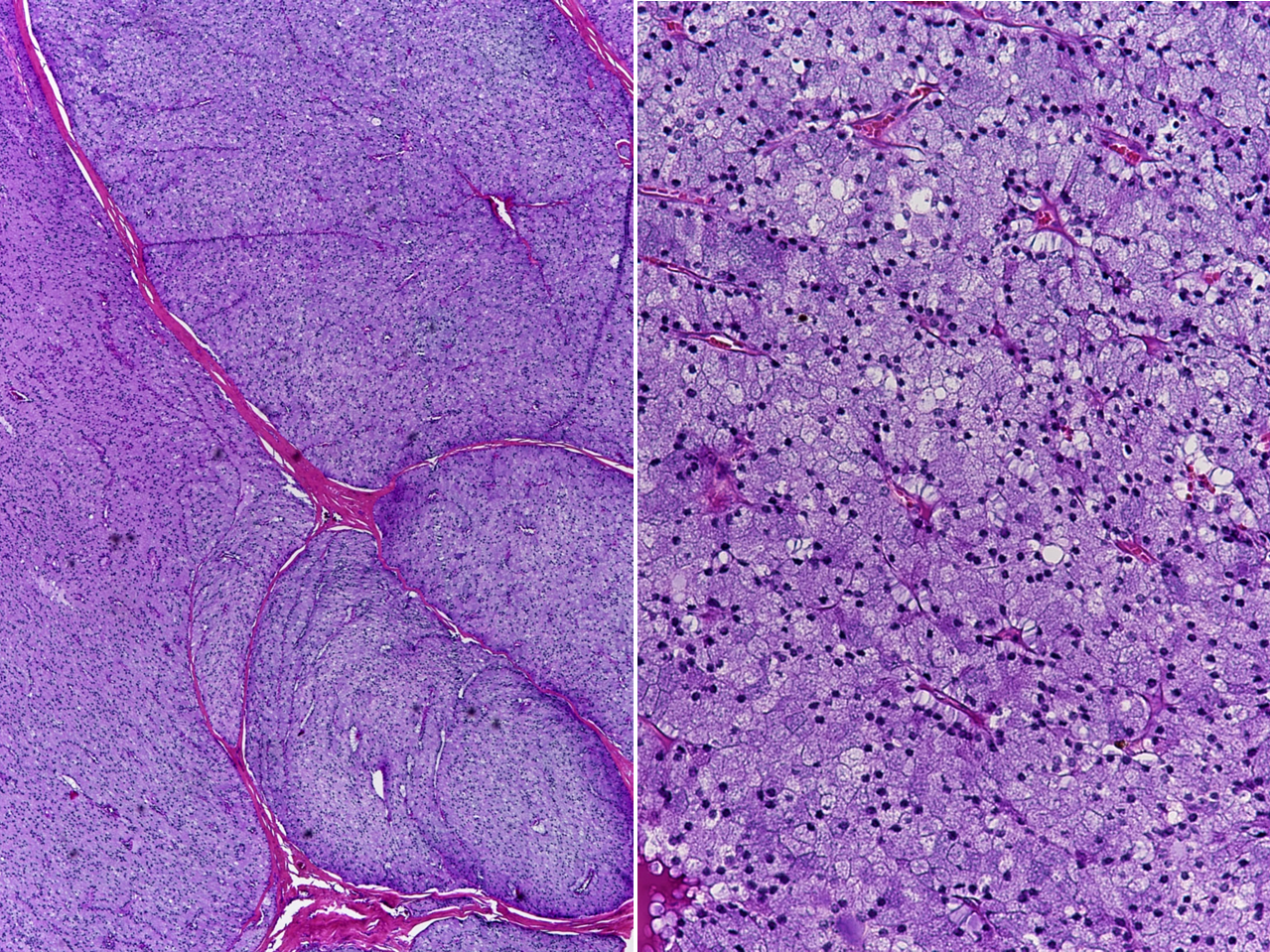

Supplement: Supplementary file 3 — Supplementary Figure 2A [file 41379_2019_427_MOESM3_ESM.tif]

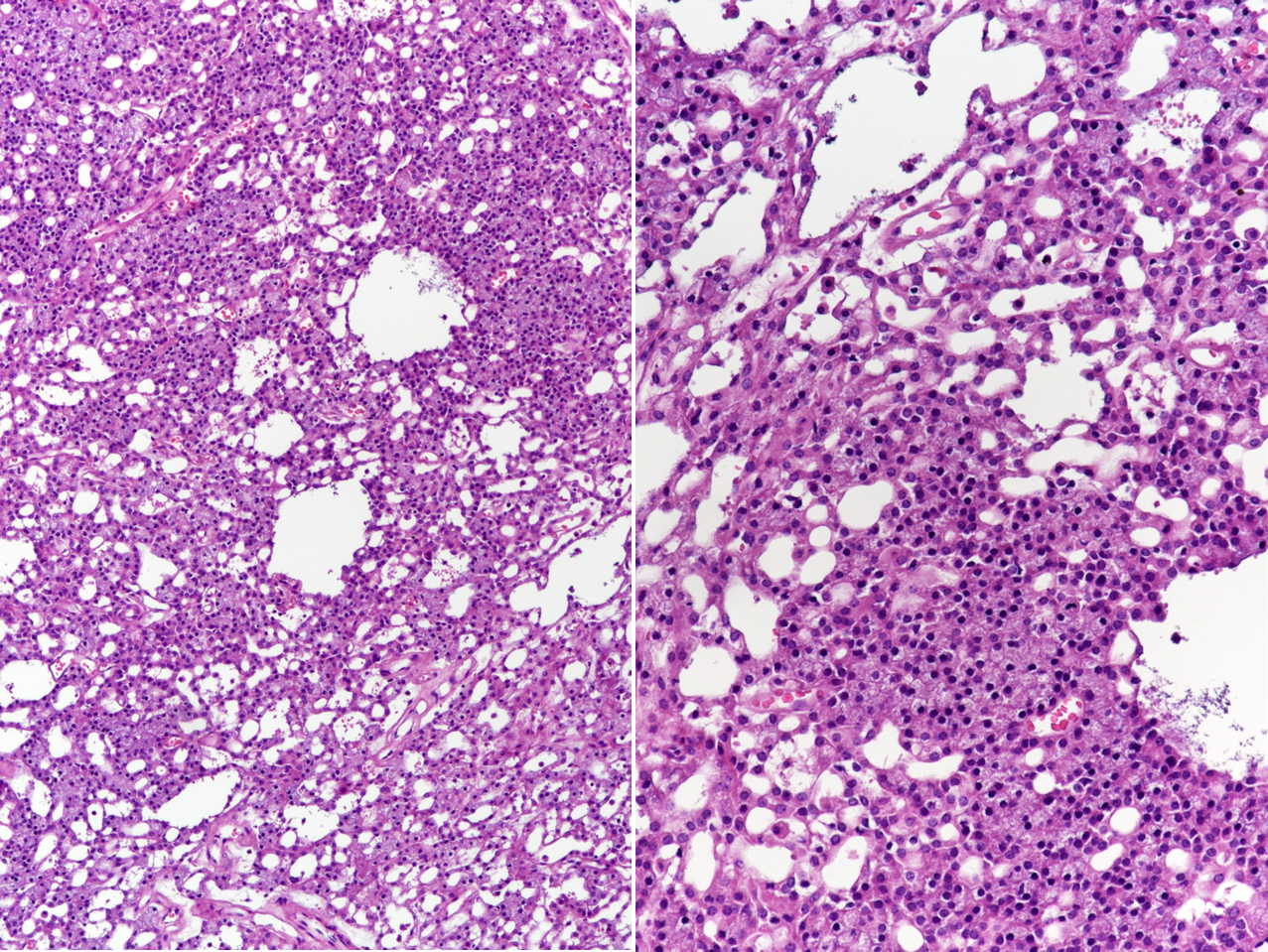

Supplement: Supplementary file 4 — Supplementary Figure 2B [file 41379_2019_427_MOESM4_ESM.tif]

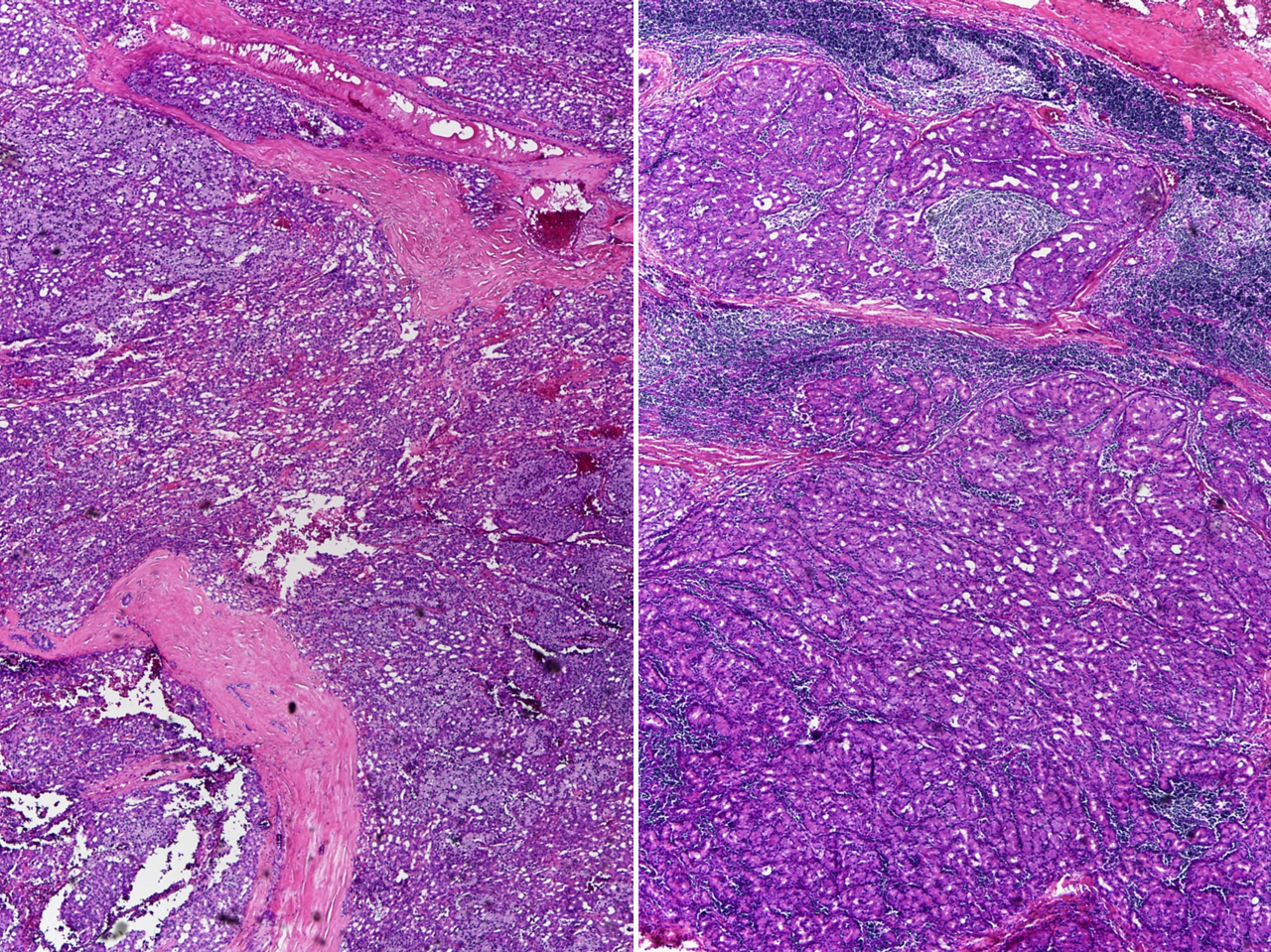

Supplement: Supplementary file 5 — Supplementary Figure 2C [file 41379_2019_427_MOESM5_ESM.tif]

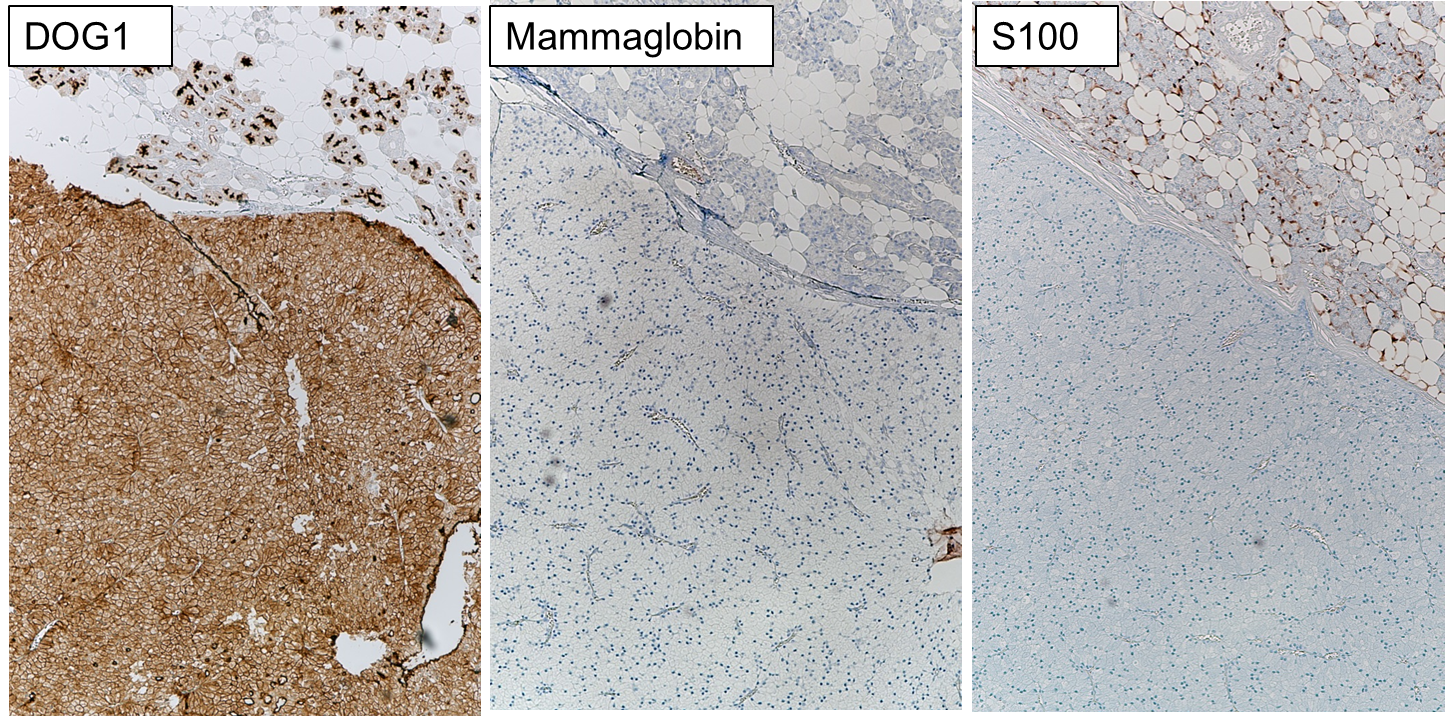

Supplement: Supplementary file 6 — Supplementary Figure 2D [file 41379_2019_427_MOESM6_ESM.tif]

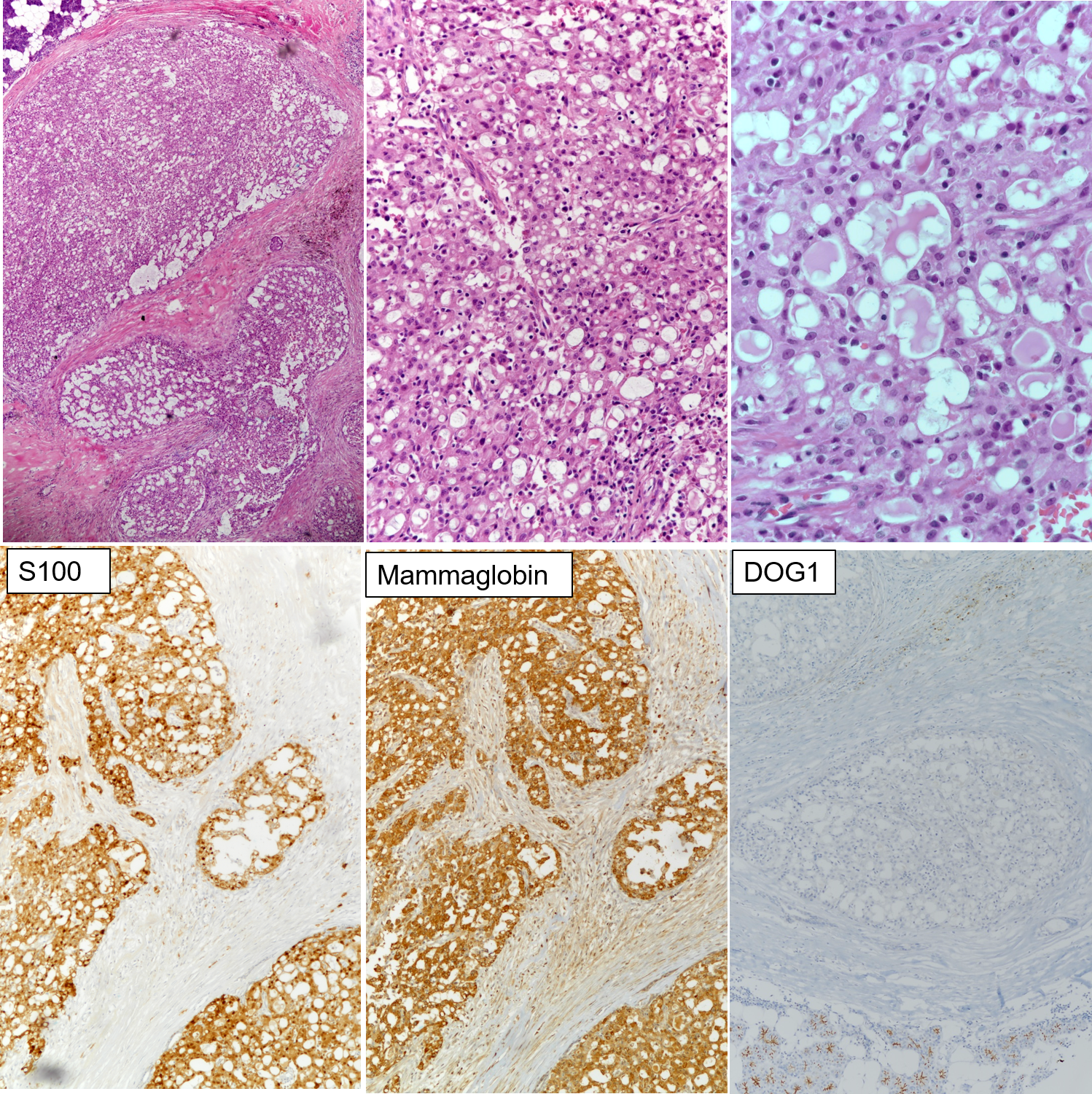

Supplement: Supplementary file 7 — Supplementary Figure 3A [file 41379_2019_427_MOESM7_ESM.tif]

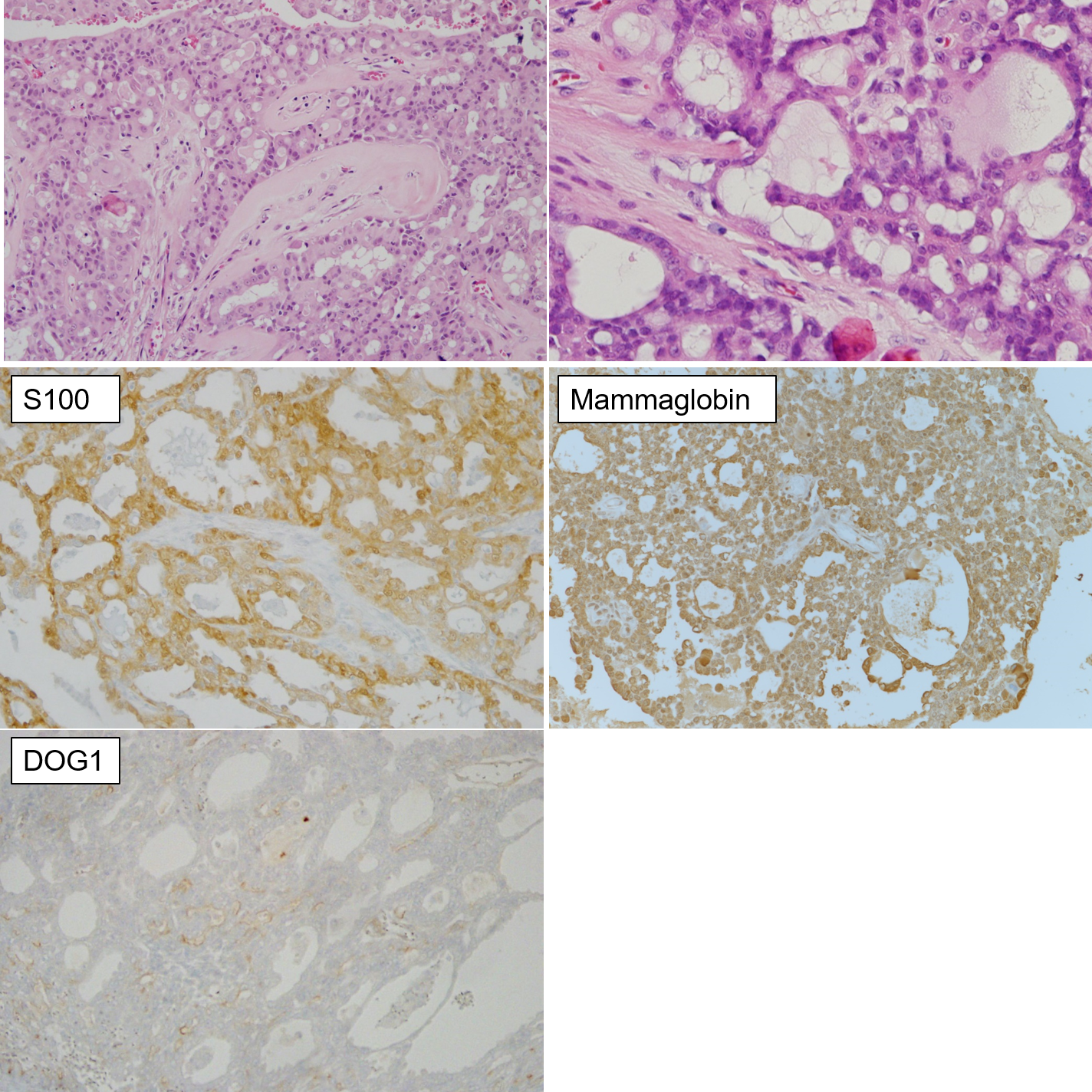

Supplement: Supplementary file 8 — Supplementary Figure 3B [file 41379_2019_427_MOESM8_ESM.tif]

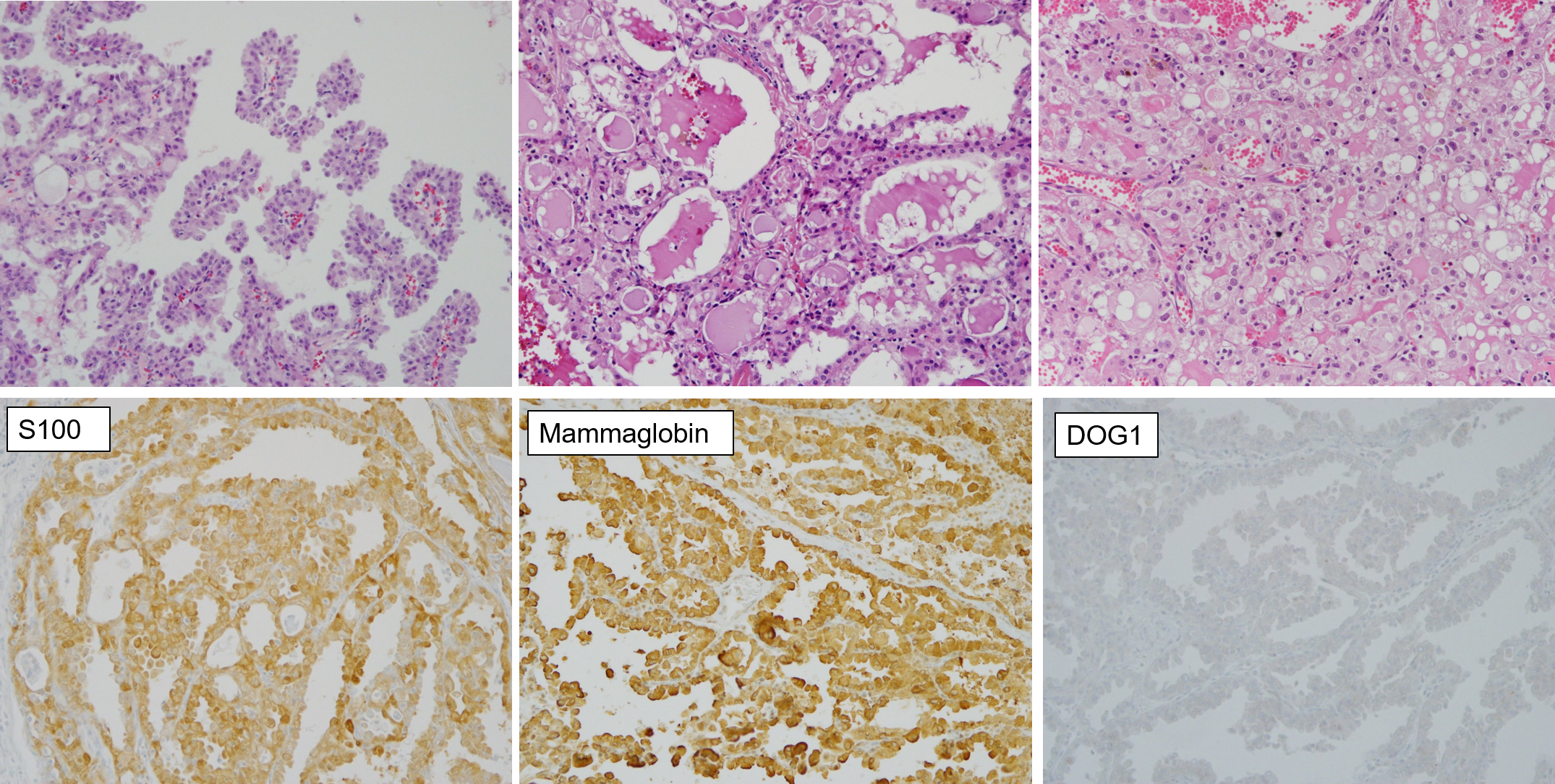

Supplement: Supplementary file 9 — Supplementary Figure 3C [file 41379_2019_427_MOESM9_ESM.tif]

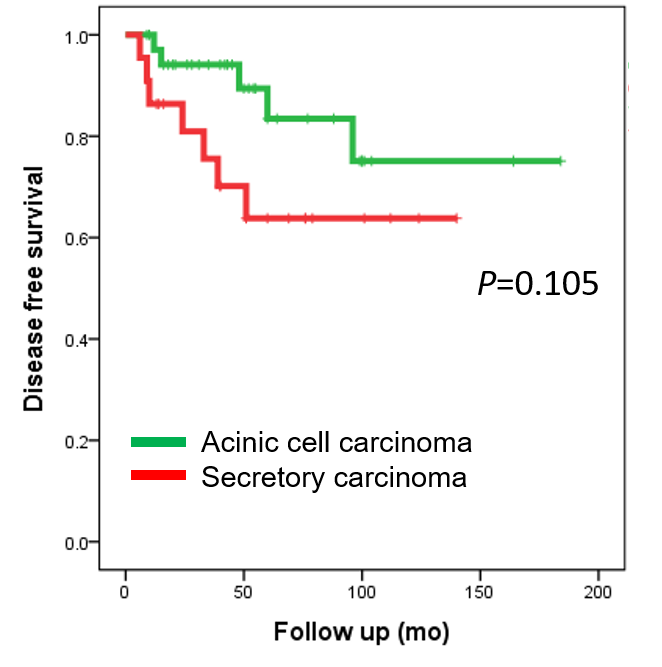

Supplement: Supplementary file 10 — Supplementary Figure 4 [file 41379_2019_427_MOESM10_ESM.tif]
