# Supplementary material for: Characterization of novel genetic alterations in salivary gland secretory carcinoma
Source: Mod Pathol. 2019 Dec 10;33(4):541–50. doi: 10.1038/s41379-019-0427-1 (PMC7113190; doi:10.1038/s41379-019-0427-1)
Supplement: Supplementary file 11 — Supplementary Table 1 [file 41379_2019_427_MOESM11_ESM.docx]

**Supplementary Table 1. Targeted Deep Sequencing Gene List**

| SNV / InDel (535 genes) | Fusion (54 genes) | Promoter(1 gene) |
| --- | --- | --- |
| *ABL1, ABL2, ABRAXAS1, ACVR1, ACVR1B, ADGRA2, AGO2, AHNAK2, AKT1, AKT2, AKT3, ALK, ALOX12B, AMER1, ANKRD11, APC, AR, ARAF, ARFRP1, ARID1A,ARID1B, ARID2, ARID5B, ASXL1, ASXL2, ATM, ATR, ATRX, AURKA, AURKB, AXIN1, AXIN2, AXL, B2M, BABAM1, BAP1, BARD1, BBC3, BCL10, BCL2, BCL2L1,BCL2L11, BCL2L2, BCL6, BCOR, BCORL1, BCR, BIRC3, BLM, BMPR1A, BRAF, BRCA1, BRCA2, BRD4, BRIP1, BTG1, BTK, CALR, CARD11, CARM1, CASP8, CBFB,CBL, CCND1, CCND2, CCND3, CCNE1, CCNQ, CD274, CD276, CD79A, CD79B, CDC42, CDC73, CDH1, CDK12, CDK4, CDK6, CDK8, CDKN1A, CDKN1B, CDKN2A,CDKN2B, CDKN2C, CEBPA, CENPA, CHD2, CHD4, CHEK1, CHEK2, CIC, COP1, CREBBP, CRKL, CRLF2, CSDE1, CSF1R, CSF3R, CTCF, CTLA4, CTNNA1,CTNNB1, CUL3, CXCR4, CYLD, CYSLTR2, DAXX, DCUN1D1, DDR2, DICER1, DIS3, DNAJB1, DNMT1, DNMT3A, DNMT3B, DOT1L, DROSHA, DUSP4, E2F3, EED,EGFL7, EGFR, EIF1AX, EIF4A2, EIF4E, ELF3, ELOC, EMSY, EP300, EPAS1, EPCAM, EPHA3, EPHA5, EPHA7, EPHB1, ERBB2, ERBB3, ERBB4, ERCC2, ERCC3,ERCC4, ERCC5, ERF, ERG, ERRFI1, ESR1, ETV1, ETV4, ETV5, ETV6, EWSR1, EZH1, EZH2, FAM46C, FANCA, FANCC, FANCD2, FANCE, FANCF, FANCG, FANCL,FAS, FAT1, FBXW7, FGF10, FGF14, FGF19, FGF23, FGF3, FGF4, FGF6, FGFR1, FGFR2, FGFR3, FGFR4, FH, FLCN, FLT1, FLT3, FLT4, FOXA1, FOXL2, FOXO1,FOXP1, FRS2, FUBP1, FYN, GABRA6, GATA1, GATA2, GATA3, GATA4, GATA6, GID4, GLI1, GNA11, GNA13, GNAQ, GNAS, GPS2, GREM1, GRIN2A, GRM3, GSK3B,H3F3A, H3F3B, H3F3C, HGF, HIST1H1C, HIST1H2BD, HIST1H3A, HIST1H3B, HIST1H3C, HIST1H3D, HIST1H3E, HIST1H3F, HIST1H3G, HIST1H3H, HIST1H3I,HIST1H3J, HIST2H3A, HIST2H3C, HIST2H3D, HIST3H3, HLA-A, HLA-B, HNF1A, HOXB13, HRAS, HSD3B1, HSP90AA1, ICOSLG, ID3, IDH1, IDH2, IFNGR1, IGF1,IGF1R, IGF2, IKBKE, IKZF1, IL10, IL7R, INHA, INHBA, INPP4A, INPP4B, INPPL1, INSR, IRF2, IRF4, IRS1, IRS2, JAK1, JAK2, JAK3, JUN, KAT6A, KDM5A, KDM5C,KDM6A, KDR, KEAP1, KEL, KIT, KLF4, KLHL6, KMT2A, KMT2B, KMT2C, KMT2D, KMT5A, KNSTRN, KRAS, LATS1, LATS2, LMO1, LRP1B, LYN, LZTR1, MAGI2,MALT1, MAP2K1, MAP2K2, MAP2K4, MAP3K1, MAP3K13, MAP3K14, MAPK1, MAPK3, MAPKAP1, MAX, MCL1, MDC1, MDM2, MDM4, MED12, MEF2B, MEN1, MET,MGA, MITF, MLH1, MPL, MRE11, MSH2, MSH3, MSH6, MSI1, MSI2, MST1, MST1R, MTOR, MUTYH, MYB, MYC, MYCL, MYCN, MYD88, MYOD1, NBN, NCOA3,NCOR1, NEGR1, NF1, NF2, NFE2L2, NFKBIA, NKX2-1, NKX3-1, NOTCH1, NOTCH2, NOTCH3, NOTCH4, NPM1, NRAS, NSD1, NSD2, NSD3, NTHL1, NTRK1, NTRK2,NTRK3, NUF2, NUP93, NUTM1, PACRG, PAK1, PAK3, PAK5, PAK6, PALB2, PARP1, PAX5, PAX8, PBRM1, PDCD1, PDCD1LG2, PDGFRA, PDGFRB, PDK1, PDPK1,PGR, PHOX2B, PIK3C2B, PIK3C2G, PIK3C3, PIK3CA, PIK3CB, PIK3CD, PIK3CG, PIK3R1, PIK3R2, PIK3R3, PIM1, PLCG2, PLK2, PMAIP1, PMS1, PMS2, PNRC1,POLD1, POLE, PPARG, PPM1D, PPP2R1A, PPP4R2, PPP6C, PRDM1, PRDM14, PREX2, PRKAR1A, PRKCI, PRKD1, PRKDC, PRSS1, PRSS8, PTCH1, PTEN,PTP4A1, PTPN11, PTPRD, PTPRS, PTPRT, QKI, RAB35, RAC1, RAC2, RAD21, RAD50, RAD51, RAD51B, RAD51C, RAD51D, RAD52, RAD54L, RAF1, RANBP2,RARA, RASA1, RB1, RBM10, RECQL, RECQL4, REL, RET, RHEB, RHOA, RICTOR, RIT1, RNF43, ROS1, RPS6KA4, RPS6KB2, RPTOR, RRAGC, RRAS, RRAS2,RTEL1, RUNX1, RUNX1T1, RXRA, RYBP, SDHA, SDHAF2, SDHB, SDHC, SDHD, SESN1, SESN2, SESN3, SETD2, SF3B1, SH2B3, SH2D1A, SHOC2, SHQ1, SLIT2,SLX4, SMAD2, SMAD3, SMAD4, SMARCA4, SMARCB1, SMARCD1, SMO, SMYD3, SNCAIP, SOCS1, SOS1, SOX10, SOX17, SOX2, SOX9, SPEN, SPOP, SPRED1,SPTA1, SRC, SRSF2, STAG2, STAT3, STAT4, STAT5A, STAT5B, STK11, STK19, STK40, SUFU, SUZ12, SYK, TAF1, TAP1, TAP2, TBX3, TCF3, TCF7L2, TEK, TERT,TET1, TET2, TGFBR1, TGFBR2, TMEM127, TMPRSS2, TNFAIP3, TNFRSF14, TOP1, TOP2A, TP53, TP53BP1, TP63, TRAF2, TRAF7, TSC1, TSC2, TSHR, U2AF1,UPF1, VEGFA, VHL, VTCN1, WISP3, WT1, WWTR1, XIAP, XPO1, XRCC2, YAP1, YES1, ZBTB2, ZFHX3, ZNF217, ZNF703, ZRSR2* | *ABL1, AKT3, ALK, AXL,BCL2, BCL6, BCR,BRAF, CCDC6,CCND1, CD74, CIITA,EGFR, EML4, ERG,ETV1, ETV6, EWSR1,EZR, FGFR1, FGFR2,FGFR3, FUS, IGH, IGK,IGL, JAK2, KIF5B,KMT2A, KRAS, LRIG3,MBIP, MET, MYC,NPM1, NTRK1, NTRK2,NTRK3, PAX5,PDGFRA, PDGFRB,PPARG, RAF1, RARA,RET, ROS1, SDC4,SLC34A2, SS18,TMPRSS2, TPM3, TRA,TRB, TRG* | *TERT* |

The Axen Cancer Master Panel (Macrogen, Seoul, South Korea) includes 535 genes for SNV/InDel; 54 genes for fusions; and 1 promoter gene. All of the experiments, as well as the genome analysis, for targeted deep sequencing were performed at Macrogen (Seoul, Republic of Korea).
