# Supplementary material for: Characterization of novel genetic alterations in salivary gland secretory carcinoma
Source: Mod Pathol. 2019 Dec 10;33(4):541–50. doi: 10.1038/s41379-019-0427-1 (PMC7113190; doi:10.1038/s41379-019-0427-1)
Supplement: Supplementary file 12 — Supplementary Table 2 [file 41379_2019_427_MOESM12_ESM.docx]

**Supplementary Table 2. Mutation List of genes**

| **Sample ID** | **Diagnosis** | **Gene** | **Exon ID** | **DNA change** | **Protein change** | **Variant information** | **Allele frequency** | **Exonic effect** | **Clinical effect** |
| --- | --- | --- | --- | --- | --- | --- | --- | --- | --- |
| 1 | secretory carcinoma | MLH1 | 12/19 | c.1151T >A | p.V384D | 3:37067240 T>A | 45.58 % ( 170 / 373 ) | missense variant | Likely pathogenic |
| 1 | secretory carcinoma | KMT 5A | 3/7 | c.290-3C>A |  | 12:123879591 C> A | 11.18 % ( 18 / 161 ) | splice region variant | Uncertain significance |
| 3 | ^a^secretory carcinoma | ST K11 | 6/10 | c.842C>T | p.P281L | 19:1221319 C> T | 48.11 % ( 127 / 264 ) | missense variant | Likely pathogenic |
| 5 | secretory carcinoma | PRSS1 | 2/5 | c.47C>T | p.A16V | 7:142458412 C> T | 9.54 % ( 39 / 409 ) | missense variant | Pathogenic |
| 6 | secretory carcinoma | MLH1 | 12/19 | c.1151T >A | p.V384D | 3:37067240 T>A | 46.75 % ( 216 / 462 ) | missense variant | Likely pathogenic |
| 6 | secretory carcinoma | KMT 5A | 3/7 | c.290-3C>A |  | 12:123879591 C> A | 18.66 % ( 25 / 134 ) | splice region variant | Uncertain significance |
| 7 | secretory carcinoma | PRSS1 | 2/5 | c.47C>T | p.A16V | 7:142458412 C> T | 3.27 % ( 13 / 398 ) | missense variant | Pathogenic |
| 7 | secretory carcinoma | PALB2 | 5/13 | c.2329G>A | p.D777N | 16:23641146 C> T | 49.01 % ( 124 / 253 ) | missense variant | Uncertain significance |
| 8 | secretory carcinoma | PRSS1 | 2/5 | c.47C>T | p.A16V | 7:142458412 C> T | 5.48 % ( 25 / 456 ) | missense variant | Pathogenic |
| 9 | secretory carcinoma | PRSS1 | 2/5 | c.47C>T | p.A16V | 7:142458412 C> T | 7.39 % ( 42 / 568 ) | missense variant | Pathogenic |
| 11 | secretory carcinoma | KMT 5A | 3/7 | c.290-3C>A |  | 12:123879591 C> A | 7.39 % ( 15 / 203 ) | splice region variant | Uncertain significance |
| 13 | secretory carcinoma | PRSS1 | 2/5 | c.47C>T | p.A16V | 7:142458412 C> T | 8.23 % ( 27 / 328 ) | missense variant | Pathogenic |
| 13 | secretory carcinoma | KMT 5A | 3/7 | c.290-3C>A |  | 12:123879591 C> A | 6.18 % ( 11 / 178 ) | splice region variant | Uncertain significance |
| 13 | secretory carcinoma | NBN | 5/16 | c.505C>T | p.R169C | 8:90990527 G> A | 53.62 % ( 185 / 345 ) | missense variant | Uncertain significance |
| 14 | secretory carcinoma | BRCA2 | 11/27 | c.6029T >G | p.V2010G | 13:32914521 T > G | 46.19 % ( 218 / 472 ) | missense variant | Uncertain significance |
| 16 | secretory carcinoma | APC | 10/16 | c.1276G>T | p.A426S | 5:112155005 G> T | 47.28 % ( 269 / 569 ) | missense variant | Uncertain significance |
| 17 | secretory carcinoma | PRSS1 | 2/5 | c.47C>T | p.A16V | 7:142458412 C> T | 5.16 % ( 27 / 523 ) | missense variant | Pathogenic |
| 17 | secretory carcinoma | MUT YH | 10/15 | c.934-2A>G |  | 1:45797760 T > C | 48.59 % ( 172 / 354 ) | splice acceptor variant | Likely pathogenic |
| 17 | secretory carcinoma | RET | 15/20 | c.2611G>A | p.V871I | 10:43615532 G> A | 47.22 % ( 119 / 252 ) | missense variant | Uncertain significance |
| 18 | secretory carcinoma | BRIP1 | 5/20 | c.485G>A | p.R162Q | 17:59926512 C> T | 45.96 % ( 205 / 446 ) | missense variant | Uncertain significance |
| 19 | ^a^secretory carcinoma | PRSS1 | 2/5 | c.47C>T | p.A16V | 7:142458412 C> T | 9.37 % ( 37 / 395 ) | missense variant | Pathogenic |
| 20 | secretory carcinoma | PRSS1 | 2/5 | c.47C>T | p.A16V | 7:142458412 C> T | 5.12 % ( 34 / 664 ) | missense variant | Pathogenic |
| 21 | secretory carcinoma | PALB2 | 5/13 | c.2329G>A | p.D777N | 16:23641146 C> T | 41.42 % ( 345 / 833 ) | missense variant | Uncertain significance |
| 21 | secretory carcinoma | BLM | 12/22 | c.2515A>G | p.K839E | 15:91312776 A> G | 49.33 % ( 148 / 300 ) | missense variant e | Uncertain significanc |
| 22 | secretory carcinoma | KMT 5A | 3/7 | c.290-3C>A |  | 12:123879591 C> A | 10.40 % ( 26 / 250 ) | splice region variant | Uncertain significance |

^a^ Cases No.3 and No.19 were of *ETV6* translocation-negative secretory carcinoma.
