# Supplementary material for: Characterization of novel genetic alterations in salivary gland secretory carcinoma
Source: Mod Pathol. 2019 Dec 10;33(4):541–50. doi: 10.1038/s41379-019-0427-1 (PMC7113190; doi:10.1038/s41379-019-0427-1)
Supplement: Supplementary file 13 — Supplementary Table 3 [file 41379_2019_427_MOESM13_ESM.docx]

**Supplementary Table 3. RNA gene fusion analysis**

| Sample ID | Diagnosis | Gene A | Gene B | Gene A Breakpoint | Gene B Breakpoint | Gene A Detail | Gene B Detail | Span reads | Split reads | Total reads |
| --- | --- | --- | --- | --- | --- | --- | --- | --- | --- | --- |
| 1 | secretory carcinoma | *ETV6* | *NTRK3* | chr12:12022900 | chr15:88483984 | NM_001987.ETV6.exon5 | NM_002530.NTRK3.exon15 | 36 | 257 | 293 |
| 4 | secretory carcinoma | *ETV6* | *NTRK3* | chr12:12022900 | chr15:88524590 | NM_001987.ETV6.exon5 | NM_002530.NTRK3.exon15 | 217 | 428 | 645 |
| 6 | secretory carcinoma | *LPP* | *KMT2A* | chr3:188537030 | chr11:118326273 | NM_001167672.LPP.intron7\|NM_005578.LPP.intron8\|NM _001167671.LPP.intron8 | NM_005933.KMT2A.intron1\|NM_001197104.KMT2A.intr on1 | 1 | 4 | 5 |
| 8 | secretory carcinoma | *ETV6* | *NTRK3* | chr12:12022900 | chr15:88483984 | NM_001987.ETV6.exon5 | NM_002530.NTRK3.exon15 | 279 | 122 | 401 |
| 8 | secretory carcinoma | *ETV6* | *NTRK3* | chr12:12022900 | chr15:88524590 | NM_001987.ETV6.exon5 | NM_001007156.NTRK3.exon15 | 251 | 144 | 395 |
| 8 | secretory carcinoma | *ETV6* | *NTRK3* | chr12:12022901 | chr15:88522692 | NM_001987.ETV6.exon5 | NM_001007156.NTRK3.exon15 | 352 | 43 | 395 |
| 11 | secretory carcinoma | *NTRK2* | *ETV6* | chr9:87541009 | chr12:11942277 | NM_006180.NTRK2.intron16\|NM_001018064.NTRK2.int ron12 | NM_001987.ETV6.intron2 | 0 | 4 | 4 |
| 12 | secretory carcinoma | *ETV6* | *PAX5* | chr12:11953482 | chr9:36846933 | NM_001987.ETV6.intron2 | NM_001280548.PAX5.intron8\|NM_001280553.PAX5.intr on7 | 0 | 4 | 4 |
| 16 | secretory carcinoma | *QKI* | *NTRK2* | chr6:163877084 | chr9:87472987 | NM_006775.QKI.intron2\|NM_206855.QKI.intron2\|NM_00 1301085.QKI.intron2\|NM_206853.QKI.intron2\|NM_2068 54.QKI.intron2 | NM_001018065.NTRK2.intron12\|NM_006180.NTRK2.int ron14 | 0 | 4 | 4 |
| 17 | secretory carcinoma | *ABL1* | *ETV6* | chr9:133698177 | chr12:11896807 | NM_007313.ABL1.intron1 | NM_001987.ETV6.intron1 | 0 | 4 | 4 |
| 19 | ^a^secretory carcinoma | *NUP214* | *ABL1* | chr9:134100289 | chr9:133658713 | NM_005085.NUP214.intron32 | NM_007313.ABL1.intron1 | 1 | 4 | 5 |
| 19 | secretory carcinoma | *KMT2A* | *PDS5A* | chr11:118354696 | chr4:39931450 | NM_005933.KMT2A.intron8\|NM_001197104.KMT2A.intr on8 | NM_001100399.PDS5A.intron2\|NM_001100400.PDS5A.i ntron2 | 0 | 4 | 4 |
| 20 | secretory carcinoma | *NTRK3* | *ETV6* | chr15:88576088 | chr12:12037379 | NM_002530.NTRK3.exon15 | NM_001987.ETV6.exon5 | 2 | 162 | 164 |
| 22 | secretory carcinoma | *BRAF* | *MKRN1* | chr7:140497134 | chr7:140156079 | NM_004333.BRAF.intron7 | NM_013446.MKRN1.intron5\|NR_117084.MKRN1.intron3 | 0 | 4 | 4 |

^a^ Case No.19 was of *ETV6* translocation-negative secretory carcinoma (SC).
